# Prognostic Factors for Postoperative Bleeding Complications and Prolonged Intensive Care after Percutaneous Hepatic Chemosaturation Procedures with Melphalan

**DOI:** 10.3390/cancers15153776

**Published:** 2023-07-25

**Authors:** Manuel Florian Struck, Robert Werdehausen, Holger Kirsten, Holger Gössmann, Rhea Veelken, Florian van Bömmel, Sebastian Stehr, Timm Denecke, Sebastian Ebel

**Affiliations:** 1 Department of Anesthesiology and Intensive Care Medicine, University Hospital Leipzig, Liebigstr. 20, 04103 Leipzig, Germany; robert.werdehausen@medizin.uni-leipzig.de (R.W.); sebastian.stehr@medizin.uni-leipzig.de (S.S.); 2 Institute for Medical Statistics, Informatics and Epidemiology, Medical Faculty, University of Leipzig, 04107 Leipzig, Germany; holger.kirsten@imise.uni-leipzig.de; 3 Department of Diagnostic and Interventional Radiology, University Hospital Leipzig, Liebigstr. 20, 04103 Leipzig, Germany; holger.goessmann@medizin.uni-leipzig.de (H.G.); timm.denecke@medizin.uni-leipzig.de (T.D.); sebastian.ebel@medizin.uni-leipzig.de (S.E.); 4 Division of Hepatology, Department of Gastroenterology, University Hospital Leipzig, Liebigstr. 20, 04103 Leipzig, Germany; rhea.veelken@medizin.uni-leipzig.de (R.V.); florian.vanboemmel@medizin.uni-leipzig.de (F.v.B.)

**Keywords:** liver metastases, primary liver tumors, chemosaturation, chemofiltration, melphalan, percutaneous hepatic perfusion, heparin, protamine, bleeding

## Abstract

**Simple Summary:**

Percutaneous hepatic melphalan perfusion (chemosaturation) is a treatment option in patients with inoperable liver metastases which is associated with considerable procedural challenges, especially hemodynamic depression, due to a reduced preload and impaired coagulation caused by the use of heparin. Studies on factors that contribute to bleeding complications and a prolonged intensive care unit length of stay are not available. In this retrospective analysis, we found that high perioperative infusion volumes and the omission of heparin reversal with protamine were associated with postoperative bleeding complications, while high infusion volumes also contributed to a length of stay in the intensive care unit of more than one day, which usually is not required. Furthermore, protamine use was not significantly associated with anaphylactic or thromboembolic complications. Our findings suggest a restrictive perioperative infusion regime and support the use of postoperative protamine for heparin reversal in chemosaturation procedures.

**Abstract:**

Percutaneous hepatic melphalan perfusion (chemosaturation) in patients with liver metastases is known to be associated with procedure-related hemodynamic depression and coagulation impairment, which may cause bleeding complications and/or a prolonged intensive care unit length of stay (ICU LOS). We retrospectively analyzed possible predictive factors for bleeding complications and an ICU LOS > 1 d in a cohort of 31 patients undergoing 90 chemosaturation procedures. Using a multivariable mixed-model approach, we identified the amount of perioperative fluid volume (OR 12.0, 95% CI 2.3–60.0, *p* = 0.003) and protamine (OR 0.065, 95% CI 0.007–0.55, *p* = 0.012) to be associated with bleeding complications. Furthermore, the amount of perioperative fluid volume was associated with an ICU LOS > 1 d (OR 5.2, 95% CI 1.4–19.0, *p* = 0.011). Heparin dosage, melphalan dosage, extracorporeal circulation time, and noradrenaline dosage had no significant effects on outcomes. Protamine use was not associated with anaphylactic or thromboembolic complications. Despite the limited sample size, these results suggest a restrictive perioperative fluid regime to be beneficial, and support the use of protamine for heparin reversal after chemosaturation procedures. Further prospective randomized trials are needed to confirm these findings.

## 1. Introduction

**Figure 1:**
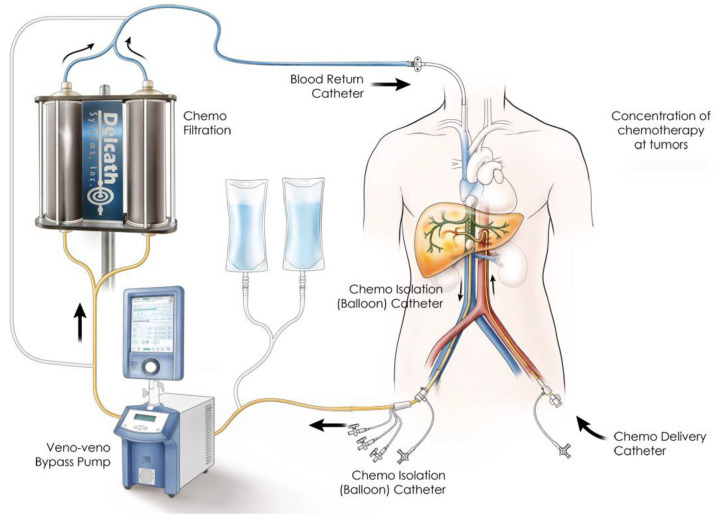
Schematic overview of the percutaneous hepatic melphalan perfusion setup (with permission from Delcath systems, Inc., Queensbury, NY, USA).

Percutaneous hepatic melphalan perfusion (chemosaturation) is a treatment option for patients with inoperable liver metastases. It may be a promising option particularly for liver metastases of ocular melanomas, which are known for their invasive growth pattern, but also for hepatocellular carcinoma, intrahepatic cholangiocarcinoma, and other carcinomas [1,2,3,4]. To achieve selective perfusion of melphalan via the hepatic arteries, a double-balloon catheter placed in the inferior cava vein is required to aspirate venous blood through an extracorporeal filtration system by pump circulation, which is then retransfused through a sheath into the internal jugular vein (Figure 1) [5]. The procedure takes 2–3 h and is usually associated with considerable hemodynamic depression, particularly when inflating the balloons, as well as during the initiation of the extracorporeal perfusion through the chemofilters and during the administration of melphalan. These steps require critical attention and communication from the interventional team when using crystalloid infusions and vasopressors to maintain acceptable hemodynamic conditions. Although the manufacturer of the chemosaturation system recommends vasopressor-based management rather than using high infusion volumes, there is only scarce data showing that infusion volumes relevantly contribute to outcomes [5].

Extracorporeal chemofiltration using blood pump circulation requires anticoagulation with an estimated activated clotting time (ACT) of >400 s to maintain functionality and prevent clotting of the filter system. After a bolus administration of heparin, frequent ACT measurements are performed throughout the procedure to maintain the estimated ACT. After the discontinuation of the bypass, reversal of the heparin effect may be provided by protamine. Additionally, the chemosaturation procedure itself is associated with further coagulation disorders (i.e., thrombopenia and impaired prothrombin time), and there is a risk for postoperative bleeding complications [1,2,3,4,5,6,7,8,9]. Thus, the central venous sheaths remain in place until the coagulation has returned to normal levels, usually within one day in the intensive care unit. Protamine was commonly used to reverse the heparin effect until cases of severe thromboembolic complications (e.g., myocardial infarction, stroke, and pulmonary embolism) were reported [2,4,6,7,8]. Some centers, including ours, have changed their practice of heparin reversal to a case-by-case decision [4,9]. The extent to which postoperative complications are dependent on heparin reversal by protamine has not yet been sufficiently explored.

The purpose of this analysis was to identify factors that contribute to bleeding complications and a prolonged intensive care unit length of stay (ICU LOS).

## 2. Materials and Methods

After receiving approval from the ethical commission, we analyzed our cohort of patients who underwent hepatic chemosaturation procedures from 2016 to 2022; postoperative bleeding complications were defined according to Clavien Dindo classes III-IV, and a prolonged ICU LOS was defined as more than one day after chemosaturation. Investigated predictors were extracorporeal circulation pump (ECCP) time (min), melphalan dosage (mg), noradrenaline (norepinephrine) dosage (µg kg^−1^ min^−1^), crystalloid fluid volume (mL kg^−1^), heparin dosage (U kg^−1^), and protamine use (yes/no). Variables including surrogate parameters of selected predictors, e.g., mean arterial blood pressure and coagulation values (e.g., ACT and activated partial thromboplastin time (aPTT)), were not considered. Data are reported as medians and interquartile ranges (IQRs) or frequencies and relative percentages.

Data were analyzed using R 4.2.2 (R Foundation for Statistical Computing, Vienna, Austria). Mixed-effects logistic regression was performed in the framework of generalized-linear mixed models using scaled numeric predictors and applying the R package lme4 1.1.31, while regression diagnostics were supported by the DHARMa 0.4.6 package. Thereby, confidence intervals were calculated by applying the Wald z-distribution approximation, which were implemented in package parameters 0.20.2. We considered a *p*-value of <0.05 as indicative of statistically significant findings.

We used backward elimination to select the best multivariate mixed-effects logistic regression model, including a random intercept term to account for subjects contributing to our study with more than one observation. This procedure involved first including all predictors in the initial model that were significant at the level of *p* < 0.2, which was followed by the sequential removal of nonsignificant predictors from this model at the 0.05 significance level. Thereby, in each step, the nonsignificant predictor with the largest *p*-value was removed unless only predictors with *p*-values larger than 0.05 remained in the model. We assessed model fit and model assumptions, including residual plots, using the R package DHARMa 0.4.6.

Possible trends in the management of investigated predictors over time were analyzed using the Spearman correlation coefficient rho (ρ) based on the first melphalan perfusion of each patient. All data and calculation codes of this study are publicly available without restrictions.

## 3. Results

### 3.1. Study Cohort

The study cohort consisted of 31 patients who underwent 90 chemosaturation procedures in total (Table S1). Twenty-one patients underwent repeated procedures, ranging from two to six procedures, whereas ten patients underwent only one procedure. The median (IQR) age was 61 (56–70) years, and 21 patients were female (67.7%) Underlying diseases included ocular melanoma (*n* = 17), intrahepatic cholangiocarcinoma (*n* = 8), hepatocellular carcinoma (*n* = 2), and other carcinomas (*n* = 4).

The median total crystalloid infusion volume was 5000 mL (range from 2000 to 14,000 mL), whereas 13 cases (14.4%) had volumes of ≥8000 mL, and 4 cases (4.4%) exceeded volumes of 10,000 mL. Three patients received an additional albumin infusion, two of which had crystalloid infusion volumes ≥8000 mL. The perioperative transfusion of red cells, fresh frozen plasma, and platelets was not performed.

An initial heparin bolus administration of 300–400 U/kg reached the ACT target of >400 s in 67 cases (74.4%), whereas in 23 cases (25.6%), additional heparin was required before chemofilter bypass activation was achieved. Postoperative heparin reversal with protamine was performed in 68 procedures (in 16 cases (24%), full reversal with a heparin-to-protamine ratio of 1:1, and in 52 cases (76%), reduced protamine dosages and heparin-to-protamine ratios of 4:3 (31 cases), 3:2 (10 cases), 2:1 (4 cases), and 3:1 (7 cases)), whereas in 22 procedures protamine was omitted. The characteristics of the procedures are presented in Table 1.

### 3.2. Postoperative Bleeding Complications

Postoperative bleeding complications matching class III or greater were observed in 13 cases (14.4%) (Table 2). Of these, four cases received heparin reversal using protamine (two using full 1:1 reversal, and another two using reduced protamine dosages and heparin to protamine ratios of 4:3 and 3:2), whereas nine cases received no protamine. Three cases, all of which did not receive protamine, developed critical conditions requiring re-intubation due to upper airway congestion (one in the angiography unit and two in the ICU), one of which underwent cardiopulmonary resuscitation. The latter required open surgery (decompression of a large neck hematoma and the assistance of thoracic surgery for invasive airway management), while endovascular histoacryl embolization of a small thyrocervical artery that was bleeding was applied in another case. Overall, nineteen cases remained mechanically ventilated during transport from the angiography unit to the ICU (including six cases with postoperative bleeding). For bleeding control, prolonged manual and mechanical compression of the puncture site (including FemoStop^TM^ (Abbott, Chicago, IL, USA)) was required in twelve cases, including five cases with femoral puncture site suturing. Postoperative blood transfusion due to bleeding was required in ten cases, which included red blood cells in five cases (nine units), fresh frozen plasma in six cases (sixteen units), and platelet concentrates in five cases (five units). Fibrinogen and tranexamic acid were each administered in four cases. All patients, including the one requiring cardiopulmonary resuscitation, recovered well and had no long-term sequelae. Mixed-effects logistic regression analysis revealed univariable associations of extracorporeal circulation pump time, the amount of crystalloid fluid volume, and protamine use with postoperative bleeding complications (Table 3). After multivariable adjustment, significant associations remained significant for the amount of crystalloid fluid volume and protamine use (Table 4). The heparin-to-protamine ratio showed no additional statistical effects regarding associations with postoperative bleeding complications and an intensive care unit length of stay >1 d.

According to the model, on average protamine lowered the probability of postoperative bleeding complications from approximately 15% to 1% at the median applied crystalloid fluid volume of 5000 mL. Considering the upper 95% prediction interval, the risk of bleeding complications exceeded 50% when the crystalloid fluid volume was more than 4000 mL in cases without protamine, and more than 7000 mL in cases with protamine.

### 3.3. Prolonged Intensive Care Unit Length of Stay

A prolonged ICU LOS of more than one day was observed in nine cases (10%). Our analysis revealed that postoperative bleeding complications were associated with a prolonged intensive care unit length of stay, but not vice versa. Univariable associations were seen with crystalloid fluid volume, protamine use, and extracorporeal circulation pump time. After the multivariable generalized linear mixed-effects model analysis, crystalloid fluid volume was the only significant predictor (Table 5 and Table 6).

### 3.4. Development of Predictors across the Observation Period

**Figure 2:**
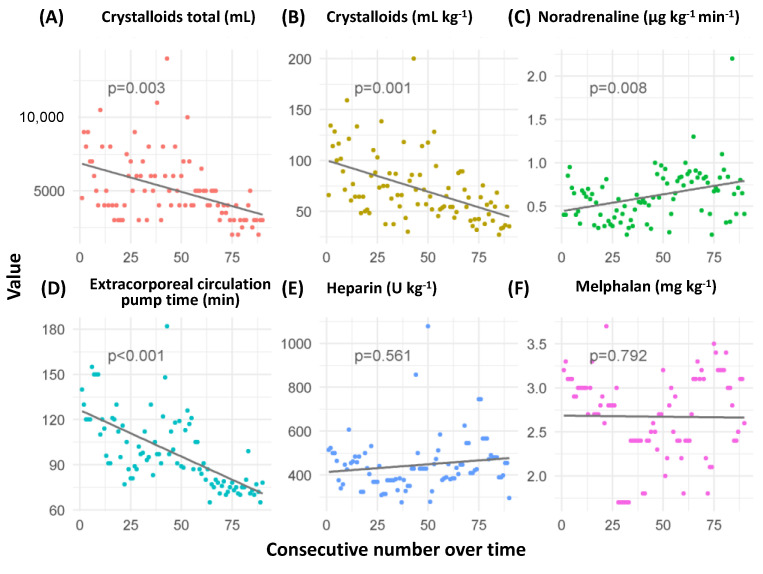
Prognostic variables across time course of the observation period years, 2016–2022. Subfigures are crystalloids volume (**A**,**B**), noradrenaline dosage (**C**), extracorporeal circulation pump time (**D**), heparin dosage (**E**), and melphalan dosage (**F**). *p*-values correspond to calculated Spearman’s rank correlation.

During the course of the observation period, the applied crystalloid fluid infusion volumes decreased considerably (total volume (ρ = −0.513, *p* = 0.003) and mL/kg (ρ = −0.567, *p* = 0.001)), while noradrenaline dosages increased (ρ = 0.466, *p* = 0.008). The extracorporeal circulation pump time decreased significantly (ρ = −0.744, *p* < 0.001). Heparin and melphalan dosages did not differ significantly across the observation period (ρ = 0.11, *p* = 0.561 and ρ = −0.05, *p* = 0.791, respectively) (Figure 2).

## 4. Discussion

Our main findings suggest that high perioperative crystalloid fluid infusion volumes and the omission of heparin reversal with protamine may be associated with postoperative bleeding complications after hepatic chemosaturation procedures. Furthermore, high infusion volumes may contribute to a prolonged ICU LOS. Adverse events regarding protamine use, particularly anaphylaxis or thromboembolic complications, were not observed. However, this should be considered with caution, since the number of analyzed procedures was limited to 90 cases. A recent dual-center study involving 256 hepatic chemosaturation procedures reported 10 thromboembolic complications, including 6 major events in 192 cases, in which full heparin reversal with protamine was performed. In 21 cases with reduced protamine dosages and 43 cases without reversal, no thromboembolic events were observed [10]. Compared with the large proportion of cases with full reversal in this study (75%), our cohort presented here consisted of only 16 cases (18%) with full reversal, which might explain the missing thromboembolic complications. As discussed later, these results suggest that partial reversal with a reduced dosage of protamine might be appropriate to avoid thromboembolic complications as well as to prevent bleeding complications. This particular property should be explored in future studies.

Although protamine was previously applied only at the discretion of the interventional team, we found it to have an independent protective effect on postoperative bleeding complications. As a result of this analysis, we have changed our strategy back to a mandatory reversal while we continue to discuss the correct protamine dose for each case individually after termination of the extracorporeal circulation. Recent studies of other endovascular procedures, particularly percutaneous coronary intervention [11,12,13], transcatheter aortic valve replacement [14], and carotid recanalization [15,16], revealed beneficial effects of protamine for heparin reversal and only low complication rates. However, protamine may cause complex interactions in the coagulation system, including procoagulatory effects, which are not fully explored yet [17,18]. This might contribute to further coagulation impairment after chemosaturation, which has to be investigated in future studies. The different half-lives of intravenous heparin (30–60 min) and protamine (5–7 min) may also influence decisions on whether to provide full reversal or only partial reversal [19,20,21,22,23,24,25]. Rebound effects after protamine dosages that are too low might be possible, and aPTT should be frequently monitored during the first postoperative hours and before transfer to the normal ward. Recent data suggest that reduced protamine dosages may be sufficient and are not be associated with further bleeding complications compared to that in full reversal [26,27]. Due to its potential to cause severe anaphylactic reactions, protamine should be infused slowly and under increased attention [28]. Immediate treatment should be available, i.e., one should have the ability to provide large infusion volumes rapidly, to administer adrenaline and noradrenaline [29].

In the literature, the hemodynamic management of common chemosaturation procedure-related hypotension using infusions and vasopressors is regarded as mandatorily needing to be performed by those with expertise [4,5,6,7]. However, there are a few studies that particularly analyzed hemodynamic management in chemosaturation procedures. In a previous evaluation of our patients, which included 53 procedures among 16 patients, or 59% of the cases presented here, we found that considerable hemodynamic affection occurred in all patients [9]. We also confirmed the findings of previous reports that fluid management that is too liberal contributed to undesired effects (e.g., edema, impairment of gas exchange, and hemodilution) [4,6]. Our present data suggest that the risk of postoperative bleeding complications is independently associated with increasing crystalloid fluid infusion volumes and that this effect is enhanced when protamine use is omitted. Studies involving hepatic surgery patients [30] and liver transplantation patients [31,32], who present with a similar pathophysiology due to intermittent clamping of the inferior cava vein, also support restrictive infusion management to reduce the risk of bleeding. Furthermore, two recent meta-analyses of randomized controlled trials revealed that goal-directed therapy approaches reduce postoperative complications, particularly when using combinations of fluids and vasopressors compared with fluids alone [33,34]. In our experience, short-timed small-volume administrations of crystalloids using rapid infusion systems are usually sufficient before balloon inflation and during initiation of the chemofilter bypass, whereas the main hemodynamic control should be based on vasopressor (e.g., noradrenaline) administration [9].

Our analysis indicated that there were no significant effects of extracorporeal circulation pump time and dosages of melphalan, noradrenaline, and heparin on the risk for both bleeding complications and a prolonged ICU LOS. Notably, pump time showed a non-significant trend which might be related to the low sample size, but it should be considered in further studies. In other populations, particularly in those who undergo cardiopulmonary bypass surgery, prolonged pump time may be a relevant prognostic factor for postoperative morbidity and bleeding complications, as it may be possibly related to complex inflammatory triggers due to the shearing forces of non-pulsatile blood flow and exposure to the artificial surfaces of the bypass circuit [35,36,37].

The present results reveal the learning process of a single center, showing a significant decrease in pump time and crystalloid fluid infusion volumes and an increase in noradrenaline dosage, while melphalan and heparin administration remained constant within the ranges of the recommended dosages. This demonstrates that performing hepatic chemosaturation procedures requires special centers with appropriate infrastructure and attending teams with professional interdisciplinary expertise. Although there are hepatic chemosaturation procedures that occur without any complications, suggesting a postoperative transfer to the recovery room or post-anesthesia care unit, we recommend an enhanced observation of the patients in the ICU at least for one night until the coagulation parameters have normalized and all large-bore vascular catheters have been safely removed. In this context, we acknowledge that angiography units are usually not based in central parts of hospitals and there may be the need for postoperative mechanical ventilation. Furthermore, our case series demonstrates that delayed complications, particularly respiratory distress due to neck swelling, may occur hours after the procedure, which warrants the ability and preparedness for emergency airway management and resuscitation measures.

### Limitations

The main limitations of this analysis are the retrospective design and the moderate number of observations. Although we used a mixed-model approach for repeated measures, including a random intercept, confounding effects cannot be excluded due to small sample sizes. Furthermore, the use of protamine for heparin reversal as well as the treatment with crystalloid fluid infusion was performed under the discretion of the attending team and was not based on a study protocol. However, we present relevant real-world data on bleeding complications and prolonged intensive care after percutaneous hepatic chemosaturation procedures, which have to be confirmed in future studies.

## 5. Conclusions

In hepatic chemosaturation procedures, high perioperative crystalloid fluid infusion volumes and the omission of heparin reversal with protamine may be associated with postoperative bleeding complications. Furthermore, high fluid infusion volumes may contribute to a prolonged ICU LOS.

## Figures and Tables

**Table 1:** Procedure characteristics.

	Value
ECCP time, min; median (IQR)	92 (78–115.5)
Melphalan, mg kg^−1^; median (IQR)	3 (2.4–3.1)
Noradrenaline, µg kg^−1^ min^−1^; median (IQR)	0.62 (0.4–0.82)
Crystalloid fluid volume total, mL; median (IQR)	5000 (3500–6000)
Crystalloid fluid volume, mL kg^−1^; median (IQR)	73 (49.8–87.8)
Heparin, U kg^−1^; median (IQR)	429 (375–484)
Heparin reversal with protamine, *n* (%)	68 (75.5)
Postoperative bleeding complication ^a^, *n* (%)	13 (14.4)
Intensive care unit length of stay > 1 d, *n* (%)	9 (10)

ECCP, extracorporeal circulation pump; ^a^ Clavien Dindo classification grades ≥ III.

**Table 2:** Overview of cases with postoperative bleeding complications.

Heparin-to-Protamine Ratio	Crystalloids (mL)	Bleeding Site	Treatment	Transfusion	ICU LOS (d)
4:3	10,500	Neck	Compression, ventilation	None	1
3:2	8000	Face	Tamponade, ventilation	1PLT	1
No protamine	7500	Neck	Re-ETI, CT, angiography	3FFP, 1 PLT, 2TXA	3
1:1	9000	Neck	Compression, ventilation	None	1
No protamine	7000	Face	Compression, ventilation	None	1
No protamine	11,000	Femoral	Compression, ventilation	1PLT, 1FFP	1
No protamine	8000	Femoral	Compression	1RBC	1
No protamine	14,000	Neck, femoral	Compression, ventilation	2RBC, 2FFP, 1PLT, 2FIB	3
No protamine	7000	Neck	Compression, ventilation	3FFP, 4FIB, 2TXA	2
No protamine	10,000	Face	Re-ETI, tamponade, ventilation	3FFP, 1TXA	2
No protamine	7000	Femoral	Compression, ventilation	1RBC, 2FIB	2
No protamine	6500	Neck	Re-ETI, CPR, Surgery, TT, ventilation	4RBC, 4FFP, 2PLT, 4FIB, 2TXA	7
1:1	3000	Femoral	Compression	1RBC	1

ETI, endotracheal intubation; CT, computed tomography; CPR, cardiopulmonary resuscitation; TT, tube thoracostomy; RBC, red blood cells; FFP, fresh frozen plasma; PLT, platelets; FIB, fibrinogen; TXA, tranexamic acid.

**Table 3:** Univariable associations with postoperative bleeding complications.

Predictor	Coefficient (SE)	Z-Value	*p*-Value
ECCP time	0.65 (0.47)	1.40	0.167
Melphalan dosage	0.096 (0.41)	0.23	0.815
Noradrenaline dosage	−0.27 (0.43)	−0.62	0.535
Crystalloid fluid volume	4.1 (2.8)	1.40	0.151
Heparin dosage	0.082 (0.39)	0.21	0.833
Protamine use	−2.7 (0.93)	−3.00	0.003

ECCP, extracorporeal circulation pump; SE, standard error. All metric predictors were scaled before analysis.

**Table 4:** Multivariable associations with postoperative bleeding complications.

Predictor	Odds Ratio (95% CI)	Z-Value	*p*-Value
Crystalloid fluid volume	12 (2.3–60)	2.9	0.003
Protamine use	0.065 (0.007–0.55)	−2.5	0.012

CI, confidence interval. Including univariable predictors with *p* < 0.2. ECCP time was nonsignificant in the multivariable generalized linear mixed-effects model. All metric predictors were scaled before analysis.

**Table 5:** Univariable associations with prolonged intensive care unit length of stay.

Predictor	Coefficient (SE)	Z-Value	*p*-Value
ECCP time	1 (0.55)	1.90	0.062
Melphalan dosage	0.47 (0.64)	0.74	0.459
Noradrenaline dosage	0.15 (0.58)	0.26	0.797
Crystalloid fluid volume	1.6 (0.62)	2.70	0.008
Heparin dosage	−0.21 (0.63)	−0.34	0.735
Protamine use	−3 (1.4)	−2.1	0.032

ECCP, extracorporeal circulation pump; SE, standard error. All metric predictors were scaled before analysis.

**Table 6:** Multivariable association with prolonged intensive care unit length of stay.

Predictor	Odds Ratio (95% CI)	Z-Value	*p*-Value
Crystalloid fluid volume	5.2 (1.5–18)	2.7	0.008

CI, confidence interval. Including univariable predictors with *p* < 0.2. After backward elimination, ECCP time and protamine use were nonsignificant in multivariable generalized linear mixed-effects models. Results of scaled crystalloid fluid volume are given.

## Data Availability

Data supporting the reported results can be found in Table S1.

## Supplementary Materials

The following supporting information can be downloaded at: https://www.mdpi.com/article/10.3390/cancers15153776/s1, Table S1: RAW_DATA_Chemosat.
